# The Coupling between Ca^2+^ Channels and the Exocytotic Ca^2+^ Sensor at Hair Cell Ribbon Synapses Varies Tonotopically along the Mature Cochlea

**DOI:** 10.1523/JNEUROSCI.2867-16.2017

**Published:** 2017-03-01

**Authors:** Stuart L. Johnson, Jennifer Olt, Soyoun Cho, Henrique von Gersdorff, Walter Marcotti

**Affiliations:** ^1^Department of Biomedical Science, University of Sheffield, Sheffield S10 2TN, United Kingdom,; ^2^Vollum Institute, Oregon Health & Science University, Portland, Oregon 97239, and; ^3^Center for Sensory Neuroscience, Boys Town National Research Hospital, Omaha, Nebraska 68131

**Keywords:** calcium channels, cochlea, exocytosis, hair cells, ribbon synapse

## Abstract

The cochlea processes auditory signals over a wide range of frequencies and intensities. However, the transfer characteristics at hair cell ribbon synapses are still poorly understood at different frequency locations along the cochlea. Using recordings from mature gerbils, we report here a surprisingly strong block of exocytosis by the slow Ca^2+^ buffer EGTA (10 mM) in basal hair cells tuned to high frequencies (∼30 kHz). In addition, using recordings from gerbil, mouse, and bullfrog auditory organs, we find that the spatial coupling between Ca^2+^ influx and exocytosis changes from nanodomain in low-frequency tuned hair cells (∼<2 kHz) to progressively more microdomain in high-frequency cells (∼>2 kHz). Hair cell synapses have thus developed remarkable frequency-dependent tuning of exocytosis: accurate low-latency encoding of onset and offset of sound intensity in the cochlea's base and submillisecond encoding of membrane receptor potential fluctuations in the apex for precise phase-locking to sound signals. We also found that synaptic vesicle pool recovery from depletion was sensitive to high concentrations of EGTA, suggesting that intracellular Ca^2+^ buffers play an important role in vesicle recruitment in both low- and high-frequency hair cells. In conclusion, our results indicate that microdomain coupling is important for exocytosis in high-frequency hair cells, suggesting a novel hypothesis for why these cells are more susceptible to sound-induced damage than low-frequency cells; high-frequency inner hair cells must have a low Ca^2+^ buffer capacity to sustain exocytosis, thus making them more prone to Ca^2+^-induced cytotoxicity.

**SIGNIFICANCE STATEMENT** In the inner ear, sensory hair cells signal reception of sound. They do this by converting the sound-induced movement of their hair bundles present at the top of these cells, into an electrical current. This current depolarizes the hair cell and triggers the calcium-induced release of the neurotransmitter glutamate that activates the postsynaptic auditory fibers. The speed and precision of this process enables the brain to perceive the vital components of sound, such as frequency and intensity. We show that the coupling strength between calcium channels and the exocytosis calcium sensor at inner hair cell synapses changes along the mammalian cochlea such that the timing and/or intensity of sound is encoded with high precision.

## Introduction

The sensory neuroepithelium of vertebrate auditory organs is tonotopically organized such that the characteristic frequency of the hair cells (the sound frequency at which they respond best) gradually changes with their position along the sensory organ. Hair cells have developed unique morphological, molecular, and biophysical features that allow them to distinguish a wide range of sound frequencies and intensities (Fettiplace and Fuchs, 1999) while maintaining submillisecond temporal precision (Matthews and Fuchs, 2010; Heil and Peterson, 2017). However, the mechanisms by which hair cell ribbon synapses ensure accurate sound encoding are still largely unknown (Fuchs, 2005; Safieddine et al., 2012). Ribbons are able to tether a large number of vesicles at the cell's presynaptic active zones, allowing them to facilitate high rates of sustained neurotransmission (Glowatzki and Fuchs, 2002; Keen and Hudspeth, 2006). Vesicle fusion at hair cell synapses is triggered by Ca^2+^ entry through Ca_V_1.3 Ca^2+^ channels (Platzer et al., 2000), clustered at the cell's presynaptic active zones (Roberts et al., 1990; Coggins and Zenisek, 2009; Frank et al., 2010), and uses otoferlin as the major Ca^2+^ sensor for exocytosis (Roux et al., 2006). However, how Ca^2+^ is able to regulate exocytosis at mature ribbon synapses is still mostly undetermined.

Spatial tightening between Ca^2+^ channels and docked vesicles improves release efficiency and is important for fast and high-fidelity neurotransmission, not only at functionally mature sensory synapses (e.g., calyx of Held) (Fedchyshyn and Wang, 2005; Leão and von Gersdorff, 2009; Chen et al., 2015), but also in the squid giant synapse (Augustine et al., 1991) and mammalian conventional synapses (Bucurenciu et al., 2008; Schmidt et al., 2013). Calcium nanodomain coupling between a few Ca^2+^ channels and the exocytotic Ca^2+^ sensor (Neher, 1998; Stanley, 2016) has also been proposed to control vesicle fusion in inner hair cells (IHCs) (Brandt et al., 2005; Wong et al., 2014). This tight coupling has the advantage of providing accurate temporal encoding for phase-locking to low-frequency tones (Rose et al., 1967; Li et al., 2014) and also allows for the synchronized release of multiple vesicles (Graydon et al., 2011), which produces large AMPA-receptor mediated EPSCs (Glowatzki and Fuchs, 2002). However, another hypothesis is that the coupling of Ca^2+^ influx and exocytosis is controlled by the cooperativity of many channels (Ca^2+^ microdomain), and it is the Ca^2+^ sensor that generates the efficient exocytosis in mature IHC ribbon synapses (Johnson et al., 2010).

We have previously investigated the effect of the fast Ca^2+^ chelator BATPA on exocytosis and found it to be comparable in apical and basal gerbil IHCs (Johnson et al., 2008). However, BAPTA chelates Ca^2+^ independently from the tightness of the coupling between the Ca^2+^ source and the sensor for vesicle fusion (Wang and Augustine, 2015). Here we used instead the “slow” Ca^2+^ chelator EGTA, which has similar affinities for Ca^2+^ as BAPTA, but a 140-fold slower ON-binding rate (Naraghi and Neher, 1997), which allows it to bind Ca^2+^ slower than the sensor for exocytosis and, as such, act as a high-pass temporal filter for Ca^2+^ (Wang and Augustine, 2015). Therefore, we investigated the effect of varying the intracellular EGTA concentration in hair cells positioned at different locations along the mammalian cochlea (mouse and gerbil) and the amphibian papilla (bullfrog), thus covering cells tuned to sound frequencies from ∼300 Hz to ∼30 kHz. We found that the coupling between the Ca^2+^ channels and the exocytotic Ca^2+^ sensor at hair cell ribbon synapses changes with high-frequency cells being more microdomain, allowing them to better encode a large dynamic range of sound intensities, whereas low-frequency cells operate via Ca^2+^ nanodomains for precise time encoding. We also found that recovery from vesicle pool depletion was slowed by high EGTA concentrations. We propose that exocytosis at mature hair cell ribbon synapses can operate via either Ca^2+^ nanodomain or microdomain depending on their location along the sensory epithelium, the function of which could be to optimize the responses of these primary auditory receptors.

## Materials and Methods

### 

#### 

##### Electrophysiology from mammalian hair cells.

IHCs from young adult gerbils of either sex (Müller, 1996) were studied in acutely dissected organs of Corti from postnatal day 18 (P18) to P60, where the day of birth is P0. Recordings were performed from IHCs positioned in the apical, middle, and basal gerbil cochlea of overlapping age range, which correspond to the *in vivo* mean characteristic frequency (CF) of ∼0.35, ∼2.5, and ∼30 kHz, respectively. Experiments were also performed on P19 to P26 mouse (of either sex) IHCs positioned in the apical coil of the cochlea with a mean CF of ∼3.0 kHz. All experiments in mice and gerbils were performed in accordance with Home Office regulations under the Animals (Scientific Procedures Act) 1986 and following approval by the University of Sheffield Ethical Review Committee.

Cochleae were dissected from gerbils and mice in normal extracellular solution (in mM) as follows: 135 NaCl, 5.8 KCl, 1.3 CaCl_2_, 0.9 MgCl_2_, 0.7 NaH_2_PO_4_, 5.6 d-glucose, 10 HEPES-NaOH. Sodium pyruvate (2 mM), amino acids, and vitamins were added from concentrates (Fisher Scientific). The pH was adjusted to 7.5 (osmolality ∼308 mmol/kg). The dissected cochlear coils were transferred to a microscope chamber containing extracellular solution and viewed using an upright microscope (Olympus BX51WI or Nikon FN1) with Nomarski DIC optics and a long working distance 60× water-immersion objective.

Gerbil and mouse recordings were performed at body temperature (34°C–37°C) using an Optopatch amplifier (Cairn Research). Patch pipettes (2–3 mΩ) were coated with surf-wax (Mr Zoggs SexWax) and contained the following (in mM): 106 Cs-glutamate, 20 CsCl, 3 MgCl_2_, 1 EGTA-CsOH, 5 Na_2_ATP, 0.3 Na_2_GTP, 5 HEPES-CsOH, 10 Na_2_-phosphocreatine, pH 7.3 (294 mmol/kg). In the experiments in which 1 mM EGTA was replaced by different EGTA concentrations (0.1, 5, and 10 mM), Cs-glutamate was adjusted to keep the osmolality constant. In a few experiments, perforated patch was used, and the pipette-filling solution contained the following (in mM): 110 Cs-aspartate, 21 CsCl, 3 MgCl_2_, 5 Na_2_ATP, 1 BAPTA, 5 HEPES-CsOH, 10 Na_2_-phosphocreatine, pH 7.3 (295 mmol/kg). The antibiotic amphotericin B (Merck Millipore) was dissolved in dry DMSO before dilution in the above intracellular solution to 120 or 240 μg/ml (Johnson et al., 2007).

Real-time changes in membrane capacitance (Δ*C*_m_) were measured as previously described (Johnson et al., 2008, 2010). Briefly, a 4 kHz sine wave of 13 mV RMS was applied to IHCs from −81 mV and was interrupted for the duration of the voltage step. The sine wave was small enough not to activate any significant membrane current because Δ*C*_m_ requires a high and constant membrane resistance (*R*_m_), which was 738 ± 61 mΩ (*n* = 87). In the experiments performed at the physiological membrane potentials (see Fig. 8), our single sine wave was sufficiently rapid to activate only a small amount of tonic *I*_Ca_, evident by the comparatively large *R*_m_ in these recordings (639 ± 77 mΩ, *n* = 20), which could possibly lead to some facilitation of vesicle release (Cho et al., 2011). The capacitance signal from the Optopatch was filtered at 250 Hz and sampled at 5 kHz. Δ*C*_m_ was measured by averaging the *C*_m_ trace over a 200 ms period following the voltage step and subtracting the prepulse baseline. Data were acquired using pClamp software (RRID:SCR_011323) and a Digidata 1440A (Molecular Devices) and analyzed with Origin 2016 (OriginLab, RRID:SCR_002815). Membrane potentials were corrected for the voltage drop across the series resistance (whole-cell recordings: apical coil IHCs, 4.8 ± 0.1 mΩ, *n* = 60; middle, 4.9 ± 0.2 mΩ, *n* = 12; basal, 5.5 ± 0.2 mΩ, *n* = 45; perforated patch recordings: apical coil IHCs, 5.2 ± 0.2 mΩ, *n* = 4; basal, 4.8 ± 0.1 mΩ, *n* = 5) and a liquid junction potential of −11 mV, measured between electrode and bath solutions. The cell membrane capacitance (*C*_m_) in whole cell was as follows: apical coil IHCs, 11.6 ± 0.2 pF, *n* = 60; middle, 11.3 ± 0.5 pF, *n* = 12; basal, 11.4 ± 0.4 mΩ, *n* = 45; *C*_m_ in perforated patch was as follows: apical coil IHCs, 10.8 ± 0.3 pF, *n* = 4; basal, 10.0 ± 0.5 mΩ, *n* = 5. The average voltage-clamp time constant (product of *R*_s_ and *C*_m_) in whole cell was 56 ± 2 μs in apical, 55 ± 2 μs in middle, and 62 ± 4 μs in basal IHCs; in perforated patch, it was 55 ± 2 μs in apical and 48 ± 2 μs in basal IHCs. Experiments were performed in the presence of 30 mM TEA and 15 mM 4-AP in the extracellular solution (Fluka, Sigma-Aldrich) to block the BK current (*I*_K,f:_ Kros et al., 1998) and delayed rectifier K^+^ currents (*I*_K,neo_ and *I*_K,s_), and linopirdine (80 μm: Tocris Bioscience) to block *I*_K,n_ (Marcotti et al., 2003).

Statistical comparisons of means were made by the two-tailed *t* test or, for multiple comparisons, ANOVA, one-way ANOVA followed by the Bonferroni test. Data are mean ± SEM. *p* < 0.05 indicates statistical significance.

##### Electrophysiology from bullfrog auditory hair cells.

Following an Oregon Health and Science University (Institutional Animal Care and Use Committee) approved animal care protocol, amphibian papillae of adult female or male bullfrogs (*Rana catesbeiana*) were carefully dissected as previously described (Keen and Hudspeth, 2006; Li et al., 2009). Semi-intact preparations of hair cells and their connecting afferent fibers were placed in a recording chamber with artificial perilymph containing the following (in mM): 95 NaCl, 2 KCl, 2 CaCl_2_, 1 MgCl_2_, 25 NaHCO_3_, 3 glucose, 1 creatine, 1 Na-pyruvate, pH adjusted to 7.3 with NaOH, and continuously bubbled with 95% O_2_ and 5% CO_2_ (osmolality 230 mmol/kg). Oxygenated artificial perilymph was perfused continuously (2–3 ml/min) during the recordings, which were performed at room temperature.

An Olympus BX51WI microscope equipped with a 60× water-immersion objective lens (Olympus) and digital CCD camera (QImaging Scientific) were used to view the preparation, and electrophysiological recordings were performed in the middle area of amphibian papillae at an average CF of ∼0.4 kHz (Li et al., 2014). All recordings were performed at room temperature using an EPC-10/2 patch-clamp amplifier and Patchmaster software (HEKA, RRID:SCR_000034). The control intracellular pipette solution contained the following (in mM): 77 Cs-gluconate, 20 CsCl, 1 MgCl_2_, 10 TEA-Cl, 10 HEPES, 2 EGTA, 3 Mg-ATP, 1 Na-GTP, and 5 Na_2_-phosphocreatine (adjusted to pH 7.3 with CsOH). The amount of Cs-gluconate was adjusted to match osmolarity of 230 mmol/kg for pipette solution containing 0.1 and 10 mM EGTA instead of 2 mM EGTA. For whole-cell recordings, patch pipettes of borosilicate glass were pulled to resistances of 6–7 mΩ for hair cells and 8–9 mΩ for afferent fibers. Hair cells were voltage-clamped with a resting membrane potential of either −60 mV or −90 mV, and afferent fibers were held at −90 mV (Cho and von Gersdorff, 2014). Membrane potentials were corrected for a liquid junction potential of 10 mV. The current signal was low-pass filtered at 5.0 kHz and sampled at 100 kHz. The averaged uncompensated series resistances in whole-cell recordings were 12.1 ± 0.2 mΩ for hair cells (*n* = 93) and 26.5 ± 1.7 mΩ for afferent fibers (*n* = 17). Δ*C*m measurements were performed under voltage clamp with the “Sine + DC” method (Lindau and Neher, 1988; Gillis, 2000) using an EPC-10/2 (HEKA) patch-clamp amplifier and Patchmaster software (HEKA). Under voltage-clamp conditions, 2 kHz sine waves were superposed on the holding potential and the resulting current response was used to calculate *C*_m_ via a Patchmaster software emulator of a lock-in amplifier (Gillis, 2000).

Data analysis was performed with Igor Pro software (WaveMetrics, RRID:SCR_000325) and Prism (GraphPad Software, RRID:SCR_002798). Statistical significance was assessed with unpaired *t* test and one-way ANOVA followed by the Bonferroni test. Data are expressed as mean ± SEM.

## Results

Whole-cell patch-clamp recordings were used to investigate Ca^2+^-dependent exocytosis in hair cells at specific CFs of the mature gerbil, mouse, and bullfrog auditory organs. Although the mouse and the frog are the most common animal models used for hearing research, they are mainly tuned to high- (mouse hearing frequency range: ∼2–100 kHz) (Ehret, 1975; Greenwood, 1990) and low- (bullfrog amphibian papilla: ∼0.15–1.2 kHz) (Lewis et al., 1982; Li et al., 2014) frequency, respectively. The advantage of the gerbil is that it has an extended low-frequency hearing range (∼0.1–60 kHz) (Müller, 1996), more similar to human hearing (∼0.02–20 kHz) (Greenwood, 1990), which should demarcate better any tonotopic differences along the spiral extension of the cochlea in a single mammalian species.

To obtain physiologically relevant data, measurements were performed at body temperature (Johnson et al., 2005, 2010; Nouvian, 2007) and using the extracellular Ca^2+^ concentration present in the perilymph surrounding the IHCs (1.3 mM) (Wangemann and Schacht, 1996). The physiological coupling between Ca^2+^ influx and the synaptic machinery was investigated from experiments in which exocytosis was recorded in the presence of different intracellular concentrations of EGTA. This enables increases in intracellular Ca^2+^ to be buffered only relatively far away from its source and thus intercept Ca^2+^ traveling within a microdomain to the Ca^2+^ sensor for exocytosis (Neher, 1998; Stanley, 2016). This is different from the action of the Ca^2+^ chelator BAPTA, which binds Ca^2+^ more rapidly than the Ca^2+^ sensor for exocytosis and as such is able to chelate Ca^2+^ independently of the tightness of the coupling between the Ca^2+^ source and the exocytotic Ca^2+^ sensor (Wang and Augustine, 2015). As such, synaptic coupling can be inferred by the different effectiveness of EGTA and BAPTA in decoupling Ca^2+^ channels from the Ca^2+^ sensor for exocytosis. Physiological processes that are prevented by BAPTA but not by EGTA are mediated by a local or nanodomain coupling, whereas those that are blocked by both imply the presence of a longer distance between the Ca^2+^ source and its sensor (microdomain) (e.g., Adler et al., 1991, Borst and Sakmann, 1996; Meinrenken et al., 2002; Fedchyshyn and Wang, 2005; Wang and Augustine, 2015).

### Frequency-dependent variation in the coupling of Ca^2+^ influx and exocytosis

**Figure 1.**
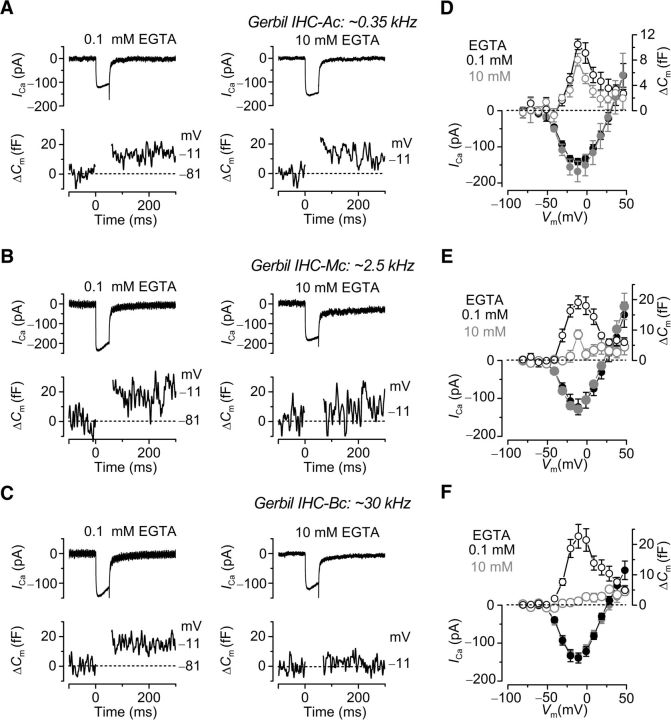
Ca^2+^ dependence of exocytosis in gerbil IHCs. ***A–C***, *I*_Ca_ and ΔC_m_ from apical (***A***: ∼0.35 kHz), middle (***B***: ∼2.5 kHz), and basal (***C***: ∼30 kHz) IHCs in the presence of 0.1 mM EGTA (left) and 10 mM EGTA (right). Recordings were obtained in response to 50 ms voltage steps from the holding potential of −81 mV to −11 mV. For clarity, only responses at −81 mV and −11 mV are shown. ***D–F***, Average peak *I–V* and Δ*C*_m_–*V* curves in apical (***D***: 0.1 mM EGTA, P20–P21, *n* = 6; 10 mM EGTA, P21–P27, *n* = 8), middle (***E***: 0.1 mM EGTA, P23–P24, *n* = 6; 10 mM EGTA, P23–P24, *n* = 7), and basal (***F***: 0.1 mM EGTA, P18–P27, *n* = 13; 10 mM EGTA, P21–P27, *n* = 10) IHCs. In this and the following figures, Ac, apical coil; Mc, middle coil; Bc, basal coil.

**Figure 2.**
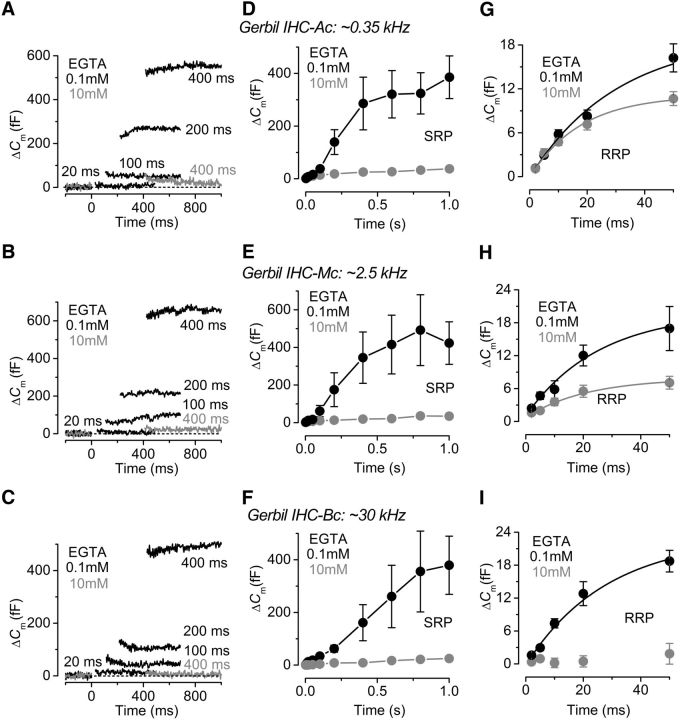
Kinetics of vesicle release in gerbil IHCs. ***A–C***, ΔC_m_ from apical (***A***), middle (***B***), and basal (***C***) IHCs in the presence of 0.1 mM EGTA (black traces) and 10 mM EGTA (gray traces). Recordings were obtained in response to voltage steps from 2 ms to 1.0 s (to ∼−11 mV) that elicit both the RRP and SRP. For clarity, only a few responses are shown. ***D–F***, Average Δ*C*_m_ obtained using the above protocol from apical (***D***: 0.1 mM EGTA, *n* = 8; 10 mM EGTA, *n* = 5), middle (***E***: 0.1 mM EGTA, *n* = 5; 10 mM EGTA, *n* = 6), and basal (***F***: 0.1 mM EGTA, P18–P20, *n* = 6; 10 mM EGTA, P21–P31, *n* = 11) IHCs revealing the SRP. ***G–I***, Isolated RRP (first 50 ms expanded from ***D–F***) approximated with single exponential functions from the average data: apical (***G***: 0.1 mM EGTA, maximum Δ*C*_m_ = 19.2 ± 5.0 fF, τ = 31 ± 12 ms; 10 mM EGTA, Δ*C*_m_ = 11.1 ± 1.0 fF, τ = 18 ± 3 ms), middle (***H***: 0.1 mM EGTA, Δ*C*_m_ = 19.9 ± 5.8 fF, τ = 25 ± 13 ms; 10 mM EGTA, Δ*C*_m_ = 7.9 ± 1.3 fF, τ = 21 ± 8 ms), and basal (***I***: 0.1 mM EGTA, Δ*C*_m_ = 23.1 ± 5.7 fF, τ = 28 ± 12 ms; 10 mM EGTA data could not be fitted because Δ*C*_m_ was almost absent). Note that the time on the x-axis in ***D***–***H*** indicates the voltage step duration.

Calcium-dependent exocytosis was measured from IHCs (P20-P27) positioned in the apical (low-frequency: CF ∼0.35 kHz), middle (CF ∼2.5 kHz), and basal (high-frequency: CF ∼30 kHz) regions of the gerbil cochlea. Calcium currents (*I*_Ca_) and corresponding Δ*C*_m_ recordings from IHCs positioned along the gerbil cochlea are shown in Figure 1. Recordings were obtained in response to 50 ms depolarizing voltage steps (holding potential of −81 mV), which allows the release of only vesicles docked at the active zones, resembling the readily releasable pool (RRP), when performing experiments using physiological 1.3 mM extracellular Ca^2+^ at body temperature (Fig. 2) (Johnson et al., 2005, 2010). The size of *I*_Ca_ was not significantly affected by the different concentrations of EGTA or by the position of the IHC along the cochlea (apical IHCs: 0.1 mM EGTA, −141 ± 9 pA, *n* = 6; 10 mM EGTA, −176 ± 18 pA, *n* = 8; middle IHCs: 0.1 mM EGTA, −122 ± 21 pA, *n* = 6; 10 mM EGTA, −129 ± 9 pA, *n* = 7; basal IHCs: 0.1 mM EGTA, −136 ± 10 pA, *n* = 13; 10 mM EGTA, −139 ± 13 pA, *n* = 10). This is consistent with previous findings showing that the size of *I*_Ca_ in apical and basal gerbil IHCs was unaffected by different concentrations of the intracellular Ca^2+^ buffer BAPTA (Johnson et al., 2008, their Fig. 5). In 0.1 mM EGTA, the peak Δ*C*_m_ was found to be not significantly different in IHCs along the cochlea (*p* = 0.09, overall one-way ANOVA). Although in apical IHCs 10 mM EGTA did not significantly affect Δ*C*_m_ (9.6 ± 1.0 fF, *n* = 8) compared with 0.1 mM EGTA (10.5 ± 0.8 fF, *n* = 6, *p* = 0.1) (Fig. 1*A*,*D*), the ability of the Ca^2+^ chelator EGTA to uncouple Ca^2+^ influx and exocytosis greatly increased toward the high-frequency region of the gerbil cochlea. In the presence of 0.1 mM intracellular EGTA, the size of the induced Δ*C*_m_ in IHCs from the middle (19.1 ± 2.1 fF, *n* = 6; Fig. 1*B*,*E*) and basal (22.7 ± 3.9 fF, *n* = 13; Fig. 1*C*,*F*) cochlear regions were significantly larger (*p* < 0.005 and *p* < 0.0001, respectively) than the values obtained when EGTA was increased to 10 mM (middle: 8.5 ± 1.5 fF, *n* = 7, Fig. 1*B*,*E*; basal: 1.3 ± 0.9 fF, *n* = 10, Fig. 1*C*,*F*). In 10 mM EGTA, Δ*C*_m_ was significantly (*p* < 0.001) smaller in basal and middle IHCs compared with apical cells. With 10 mM intracellular EGTA, the largely reduced or absent Δ*C*_m_ in middle and basal IHCs, respectively, suggests the presence of a microdomain coupling between the Ca^2+^ channels and the Ca^2+^ sensor for vesicle fusion. This finding is also supported by the fact that, although the size of Δ*C*_m_ in apical IHCs (0.1 mM EGTA: Fig. 1*D*) is comparable to that previously reported using 1 mM intracellular EGTA (50 ms voltage step) (Johnson et al., 2008), that measured in basal IHCs (Fig. 1*F*) was in most cells larger despite the similar number of synaptic ribbons per cell in the two regions (Johnson et al., 2009; Meyer et al., 2009). Because low-frequency IHCs seem to experience a nanodomain scenario, decreasing the concentration of EGTA from 1 mM (Johnson et al., 2008) to 0.1 mM (Fig. 1*D*) is unlikely to result in a different Δ*C*_m_. However, the microdomain scenario in high-frequency IHCs would allow Ca^2+^ to travel further when reducing the concentration of EGTA from 1 to 0.1 mM, and most likely able to recruit a small part of the secondary releasable pool in some IHCs (see below).

To investigate whether the vesicle pool dynamics in high EGTA also varied as a function of frequency position, we measured the rate of neurotransmitter release in gerbil IHCs (P18-P31) by measuring Δ*C*_m_ in response to depolarizing voltage steps to −11 mV of varying duration (2 ms to 1.0 s: Fig. 2; interstep interval was at least 11 s), which allowed us to investigate the emptying of different synaptic vesicle pool populations. When using 1 mM intracellular EGTA and 1.3 mM extracellular Ca^2+^, stimuli ≤50 ms reveal the RRP (see also Johnson et al., 2005, 2010). Longer steps induce the release of vesicles from a secondarily releasable pool (SRP) that is located further away from the Ca^2+^ channels (frog: Rutherford and Roberts, 2006; mouse: Moser and Beutner, 2000; gerbil: Johnson et al, 2008). In 10 mM EGTA, the release from the SRP was almost completely abolished in all IHCs investigated, regardless of their cochlear location (apical: Fig. 2*A*,*D*; middle: Fig. 2*B*,*E*; basal: Fig. 2*C*,*F*), which is also in agreement with previous reports in mice (Moser and Beutner, 2000) and lower vertebrates (Graydon et al., 2011). However, the release from the RRP was differentially affected along the gerbil cochlea. In apical low-frequency IHCs (∼0.35 kHz; Fig. 2*G*), the size of the isolated RRP in 10 mM EGTA (11.7 ± 1.2 fF, *n* = 5) was not significantly different from that obtained in 0.1 mM EGTA (18.0 ± 2.3 fF, *n* = 8, *p* = 0.07, from fits to individual IHCs), as also shown in Figure 1*D*. The initial release rate was also similar between the two recording conditions (0.1 mM EGTA: 817 ± 115 fF/s or 22,074 ± 3109 vesicles/s, *n* = 8; 10 mM EGTA: 596 ± 129 fF/s or 16,115 ± 3493 vesicles/s, *n* = 5, *p* = 0.2, from fits to individual IHCs: Fig. 2*G*). However, compared with 0.1 mM EGTA, 10 mM EGTA largely reduced the release from the RRP in middle-coil IHCs (middle ∼2.5 kHz: 0.1 mM EGTA, 20.2 ± 4.6 fF, *n* = 5; 10 mM EGTA, 7.1 ± 1.1 fF, *n* = 6, *p* < 0.02; Fig. 2*H*) and almost completely abolished it in basal cells (basal ∼30 kHz: 0.1 mM EGTA 22.1 ± 1.4 fF, *n* = 6; in 10 mM EGTA, the RRP could only be measured in 2 of 11 IHCs and was 2.4 ± 0.1 fF; Fig. 2*I*). As for IHCs in the apical coil, the initial release rate in middle IHCs was also similar between the two recording conditions (0.1 mM EGTA: 1000 ± 101 fF/s or 27076 ± 2754 vesicles/s, *n* = 5; 10 mM EGTA: 723 ± 141 fF/s or 19558 ± 3708 vesicles/s, *n* = 6, *p* = 0.2, from fits to individual IHCs).

**Figure 3.**
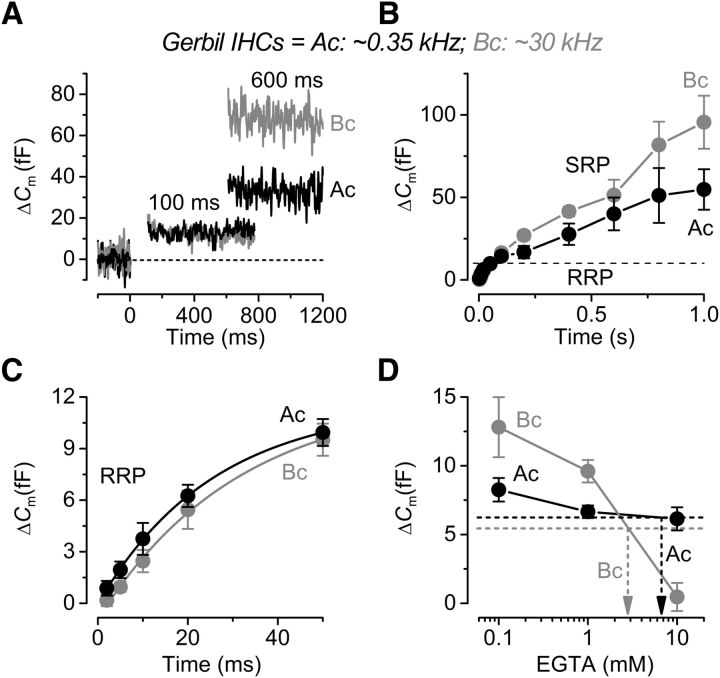
Kinetics of vesicle release in endogenous Ca^2+^ buffer from gerbil IHCs. ***A***, Δ*C*_m_ from apical (black) and basal (gray) IHCs recorded with perforated patch. Recordings were obtained as described in Figure 2. For clarity, only a few responses are shown. ***B***, Average Δ*C*_m_ from apical (P37–P52, *n* = 4) and basal (P37–P60, *n* = 5) IHCs revealing the RRP and SRP. ***C***, Isolated RRP (first 50 ms expanded from ***B***) approximated with single exponential functions from the single data (apical, maximum Δ*C*_m_ = 11.6 ± 1.8 fF, τ = 26 ± 10 ms; basal, Δ*C*_m_ = 12.2 ± 3.2 fF, τ = 31 ± 17 ms). ***D***, The perforated-patch values of Δ*C*_m_ at 20 ms, a value that is well within the range of the RRP, were extrapolated (dotted lines) to those obtained using different EGTA concentrations (data from Fig. 2). The 1 mM EGTA data are from Johnson et al. (2008). Note that the time on the x-axis in ***B*** and ***C*** indicates the voltage step duration.

Using perforated-patch recordings that preserve the endogenous intracellular Ca^2+^ buffering, we found that the pool sizes and release kinetics of the RRP and SRP were comparable between apical and basal cells in these physiological conditions (Fig. 3*A–C*). We have previously shown that the endogenous buffer concentration was similar between low- and high-frequency gerbil IHCs when expressed as an equivalent BAPTA concentration (Johnson et al., 2008). However, when the Δ*C*_m_ values obtained in perforated patch were extrapolated to those obtained using different concentrations of EGTA (Fig. 3*D*), they revealed a higher sensitivity to Ca^2+^ buffering in high-frequency IHCs (∼2.9 mM) compared with low-frequency cells (∼6.6 mM) (Fig. 3*D*). However, this is not an indication of the endogenous buffer in IHCs but provides further evidence for a different exocytotic Ca^2+^ coupling of the RRP between apical and basal cells.

### Calcium channel and vesicle coupling in high-frequency mouse IHCs

**Figure 4.**
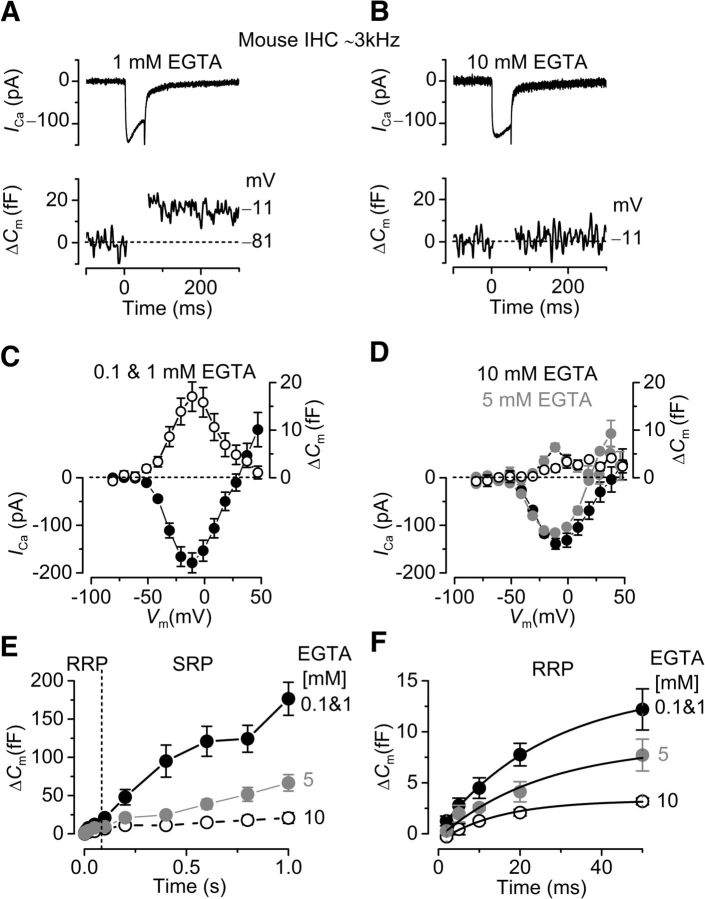
Ca^2+^ currents and Δ*C*_m_ in mouse IHCs. ***A***, ***B***, *I*_Ca_ (top panels) and Δ*C*_m_ (bottom panels) responses recorded from IHCs positioned in the apical region (CF: ∼3.0 kHz) of the mouse cochlea in the presence of low (0.1 and 1 mM) and high (10 mM) concentrations of intracellular EGTA, respectively. Recordings were obtained in response to 50 ms voltage steps from the holding potential of −81 mV to −11 mV. ***C***, ***D***, Average peak current (*I*, bottom) and capacitance (Δ*C*_m_, top) plotted as a function of membrane potential from IHCs recorded in the presence of low and high intracellular EGTA, respectively. ***E***, Average Δ*C*_m_ in response to voltage steps from 2 ms to 1.0 s (to ∼−11 mV) showing the RRP and SRP. ***F***, RRP (first 50 ms expanded from ***E***) approximated with single exponential functions for the different concentrations of EGTA used (0.1 and 1 mM: maximum Δ*C*_m_ = 16.4 ± 3.4 fF, τ = 32 ± 10 ms; 5 mM: Δ*C*_m_ = 8.2 ± 2.0 fF, τ = 21 ± 4 ms; 10 mM: Δ*C*_m_ = 3.4 ± 0.4 fF, τ = 17 ± 2 ms). The available RRP was calculated using a conversion factor of 37 aF/vesicle (Lenzi et al., 1999). Note that the time on the x-axis in ***E*** and ***F*** indicates the voltage step duration.

To confirm that the different coupling between Ca^2+^ influx and exocytosis observed in IHCs along the gerbil cochlea (Figs. 1–3) was due to the synaptic machinery being specialized to detect different frequencies, we performed experiments in the mouse and the bullfrog. Figure 4*A*, *B* shows the maximal *I*_Ca_ and the corresponding Δ*C*_m_ recorded from apical IHCs of the mouse cochlea (∼3.0 kHz) in the presence of either low (0.1 and 1 mM) or high (5 and 10 mM) concentrations of intracellular EGTA, respectively. The apical coil of the mouse cochlea has a CF in the same range to that of the middle region of the gerbil cochlea (∼2.5 kHz: Figs. 1, 2). The data from 0.1 and 1 mM EGTA (Fig. 4) were pooled together because they produced overlapping results. Responses were obtained using 50 ms depolarizing voltage steps (10 mV nominal increments) from −81 mV. The maximal size of *I*_Ca_ recorded in IHCs (P15-P26) was not significantly different between low (0.1 and 1 mM: −179 ± 21 pA, *n* = 5; Fig. 4*C*) and high EGTA (5 mM: −115 ± 7 pA, *n* = 3 or 10 mM: −139 ± 11 pA, *n* = 5; Fig. 4*D*). However, the induced Δ*C*_m_ was significantly reduced (overall: *p* < 0.002, one-way ANOVA) in the presence of 5 mM (6.4 ± 0.8 fF, *n* = 3, *p* < 0.05 post-test) or 10 mM EGTA (2.0 ± 1 fF, *n* = 5, *p* < 0.01 post-test) (Fig. 4*D*), compared with the lower concentrations (0.1 and 1 mM EGTA: 16 ± 3 fF, *n* = 6; Fig. 4*C*).

The rate of neurotransmitter release in mouse IHCs (P19-P26) was studied by measuring Δ*C*_m_ in response to depolarizing voltage steps of increasing duration (Fig. 4*E*) as described for Figure 2. Voltage steps of up to ∼50 ms (RRP) produced an increase in Δ*C*_m_ that could be approximated with a single exponential (Fig. 4*F*). However, in the presence of 5 or 10 mM intracellular EGTA, the largely reduced size of the RRP was also associated with a significantly reduced initial release rate (5 mM: 374 ± 33 fF/s or 10112 ± 877 vesicles/s, *n* = 4, *p* < 0.05 post-test; 10 mM 203 ± 36 fF/s or 5500 ± 976 vesicles/s, *n* = 3, *p* < 0.01 post-test) compared with that measured in lower EGTA concentrations (614 ± 75 fF/s or 16589 ± 2036 vesicles/s, *n* = 5: average from fit to single cells; overall: *p* < 0.005, one-way ANOVA) (Fig. 4*F*). The SRP in high EGTA was almost absent (Fig. 4*E*). Considering that the total number of ribbons per IHC is ∼20 (Brandt et al., 2005; Meyer et al., 2009; Zampini et al., 2010), the vesicle release rate per ribbon was ∼829 vesicles/s (low EGTA), 506 vesicles/s (5 mM EGTA), and 275 vesicles/s (10 mM EGTA) vesicles/s. This reduced exocytosis in mouse IHCs in the presence of high concentrations of EGTA has previously been reported in young (P12-P14) (Vincent et al., 2014) but not in more mature (>P14) (Moser and Beutner, 2000) cells using unphysiologically high extracellular Ca^2+^ (5–10 mM) and room temperature.

### Calcium channel and vesicle coupling in low-frequency tuned bullfrog hair cells

**Figure 5.**
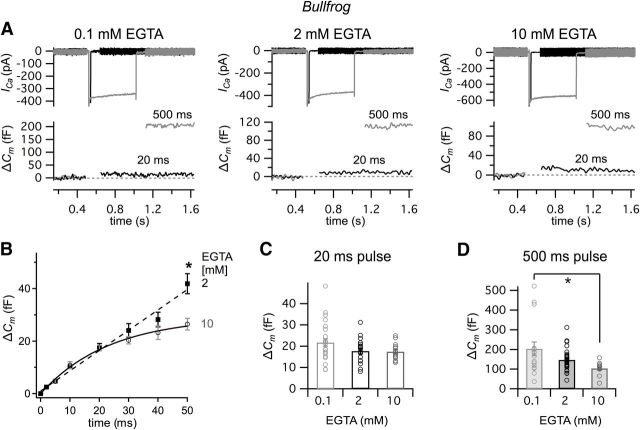
Ca^2+^ currents and Δ*C*_m_ in bullfrog hair cells tuned to ∼ 400–500 Hz sound signals. ***A***, Calcium current (*I*_Ca_) and membrane capacitance (*C*_m_) were measured while hair cells were depolarized from a holding potential of −90 mV to −30 mV for 20 ms (black) and 500 ms (gray) with 0.1 mM (left), 2 mM (middle), and 10 mM of intracellular EGTA (right). Note the change in vertical scales for the *C*_m_ data and the large Δ*C*_m_ jump (exocytosis) produced by 500 ms depolarizing pulses when 0.1 mM EGTA is used in the patch pipette internal solution. ***B***, Average Δ*C*_m_ in response to voltage steps from 2 to 50 ms with 2 mM (black) and 10 mM EGTA (gray). The depolarization of 50 ms from −90 mV to −30 mV only showed significant difference of Δ*C*_m_ between 2 and 10 mM EGTA. *p < 0.05 (unpaired *t* test). Data modified with permission from Graydon et al. (2011). ***C***, Comparison of Δ*C*_m_ in response to voltage steps of 20 ms from −90 mV to −30 mV using 0.1 mM (light gray, *n* = 27, 21.7 ± 1.7 fF), 2 mM (black, *n* = 18, 17.8 ± 1.4 fF), and 10 mM (gray, *n* = 14, 17.4 ± 1.0 fF) of EGTA. One-way ANOVA did not show significant difference (*p* = 0.098). ***D***, Comparison of Δ*C*_m_ in response to voltage steps of 500 ms pulse from −90 mV to −30 mV using 0.1 mM (light gray, *n* = 16, 203.8 ± 34.8 fF), 2 mM (black, *n* = 34, 147.3 ± 9.1 fF), and 10 mM (gray, *n* = 13, 104.1 ± 8.5 fF) of EGTA. The Δ*C*_m_ jumps in panels ***C*** and ***D*** were measured after 4 min from whole-cell break-in to allow for the full diffusion of EGTA into the hair cell. One-way ANOVA followed the Bonferroni test showed significant difference (overall: *p* = 0.006).

We next investigated *I*_Ca_ and the corresponding Δ*C*_m_ in single hair cells from a semi-intact adult bullfrog amphibian papilla preparation, which are tuned to a lower frequency range (∼400–500 Hz) (Li et al., 2014), to examine the exocytotic Ca^2+^ coupling. To measure *I*_Ca_ and Δ*C*_m_, we stimulated the hair cells with voltage-clamp step depolarizations from −90 mV to −30 mV for various durations (Fig. 5*A*). A depolarization to −30 mV elicits the peak *I*_Ca_ in bullfrog hair cells (Graydon et al., 2011). As the pulse duration increased, so did the resulting Δ*C*_m_ (Fig. 5*A*) (Li et al., 2009). We compared Δ*C*_m_ in response to depolarizing pulses with 0.1, 2 and 10 mM intracellular EGTA (Fig. 5*A*,*B*). Depolarizing pulses <50 ms did not show any significant difference in Δ*C*_m_ between 2 and 10 mM EGTA (Fig. 5*B*; unpaired *t* test, *p* < 0.05) (Graydon et al., 2011). However, Δ*C*_m_ was significantly larger for 50 ms pulses (Fig. 5*B*). Our previous study shows that a pulse <50 ms from −90 mV to −30 mV only triggers the RRP of hair cells, which includes ∼700 vesicles per hair cell, or 12 vesicles per synaptic ribbon (Graydon et al., 2011). To confirm this insensitivity of the RRP to EGTA, we compared Δ*C*_m_ in response to pulses of 20 and 500 ms with 0.1, 2, and 10 mM intracellular EGTA (Fig. 5*C*,*D*). The average Δ*C*_m_ in response to a 20 ms pulse was not significantly different with 0.1 mM (21.7 ± 1.7 fF, *n* = 21), 2 mM (17.8 ± 1.4 fF, *n* = 18), and 10 mM EGTA (17.4 ± 1.0 fF, *n* = 14, one-way ANOVA; Fig. 5*C*). In contrast, different concentrations of intracellular EGTA significantly changed the average Δ*C*_m_ in response to a 500 ms pulse (overall: *p* < 0.006, one-way ANOVA; Fig. 5*D*): Δ*C*_m_ with 0.1 mM (203.8 ± 34.8 fF, *n* = 16), which was significantly different from that with 10 mM EGTA (104.1 ± 8.5 fF, *n* = 13, *p* < 0.05, post-test; Fig. 5*A*), although the Δ*C*_m_ with 2 mM (147.3 ± 9.1 fF, *n* = 34) was not significantly different from those with 0.1 and 10 mM EGTA (post-test). In summary, our data suggest that, in stark contrast to mouse high-frequency IHCs (Fig. 4), the release of vesicles in the RRP from low-frequency hair cells is relatively insensitive to the concentration of intracellular EGTA. However, for longer depolarizing pulses of 50 and 500 ms, we do find that release is significantly reduced by 10 mM EGTA. This suggests that, during a longer depolarizing pulse, the recruitment of vesicles from a reserve pool is sensitive to global rises in intracellular free Ca^2+^ and is thus sensitive to the intracellular levels of EGTA. The RRP of amphibian papilla hair cells, which are tuned to low-frequency sound signals, is thus controlled by nanodomain Ca^2+^ coupling.

### Paired-pulse responses in gerbil IHCs and bullfrog auditory hair cells

**Figure 6.**
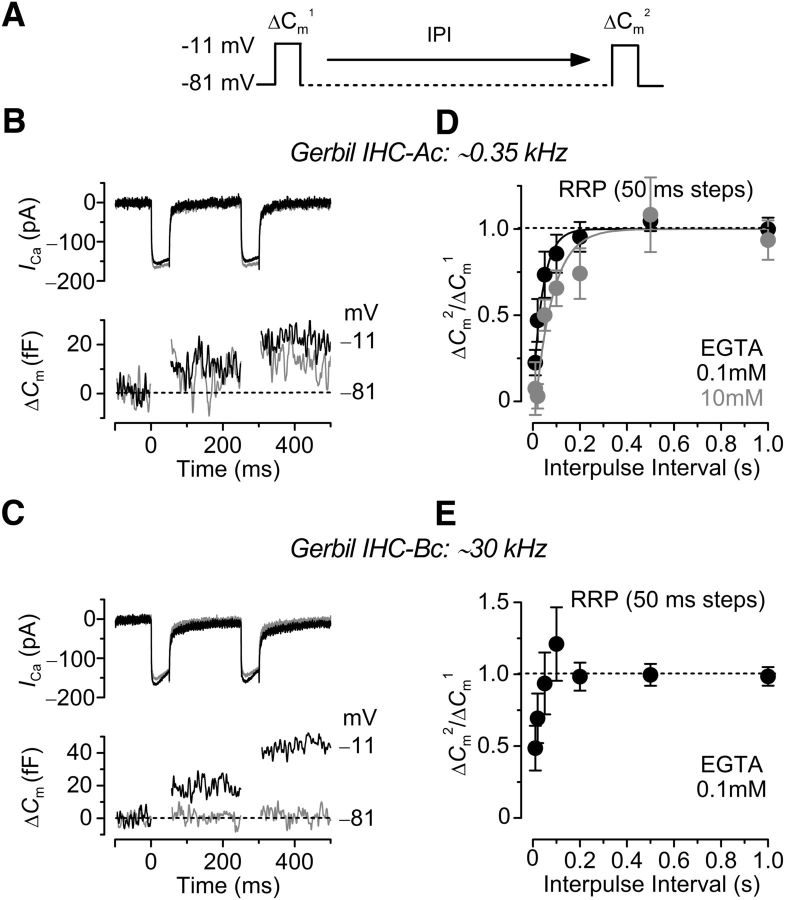
Rate of Δ*C*_m_ recovery in gerbil IHCs. ***A***, Schematic diagram of the paired-pulse protocol used to stimulate IHCs. Δ*C*_m_ was elicited in response to 50 ms depolarizing voltage steps to −11 mV (holding potential of −81 mV) at time 0 and varying the IPI (10, 20, 40, 100, 200, and 500 ms, 1 s) after the initial step. ***B***, ***C***, *I*_Ca_ and Δ*C*_m_ from apical (***B***: ∼0.35 kHz) and basal (***C***: ∼30 kHz) IHCs in the presence of 0.1 mM EGTA (black) and 10 mM EGTA (gray). ***D***, ***E***, Average Δ*C*_m_ ratio (Δ*C*_m_^2^/Δ*C*_m_^1^; ***A***) from apical (***D***) and basal (***E***) IHCs. ***D***, In apical IHCs, the data were plotted against IPI and fitted with a single exponential function (0.1 mM EGTA, τ_1_ = 42.1 ± 8.1 ms, *n* = 12; 10 mM EGTA, τ_1_ = 75.1 ± 17.1 ms, *n* = 6; significantly different at *p* < 0.0005). Basal IHCs showed an initial facilitation followed by a decline (***E***). ***E***, The data from 10 mM EGTA were omitted because Δ*C*_m_ was almost absent (see ***C***), which made it difficult to measure the Δ*C*_m_^2^/Δ*C*_m_^1^ ratio with accuracy.

We investigated possible differences in the rate of Δ*C*_m_ recovery from gerbil IHCs (P18-P27) positioned in the apical and basal cochlear regions by applying a two-pulse protocol in which cells were depolarized to −11 mV for 50 ms, which recruited the RRP, while changing the interpulse interval (IPI) from 10 ms up to 1.0 s (Fig. 6*A*). Examples of Δ*C*_m_ recorded from apical and basal IHCs using the two-pulse protocol and in the presence of either 0.1 mM EGTA or 10 mM EGTA are shown in Figure 6*B* and *C*, respectively. The average Δ*C*_m_ ratio (Δ*C*_m_^2^/Δ*C*_m_^1^: Fig. 6*A*) from apical IHCs (0.1 mM EGTA, *n* = 12; 10 mM EGTA, *n* = 6) was plotted against IPI, and the data were well approximated with a single exponential function (Fig. 6*D*). For basal IHCs, the average Δ*C*_m_ ratio in 0.1 mM EGTA showed an initial depression at short intervals and then facilitation at ∼100 ms (Fig. 6*E*) (Goutman and Glowatzki, 2011; Cho et al., 2011).

**Figure 7.**
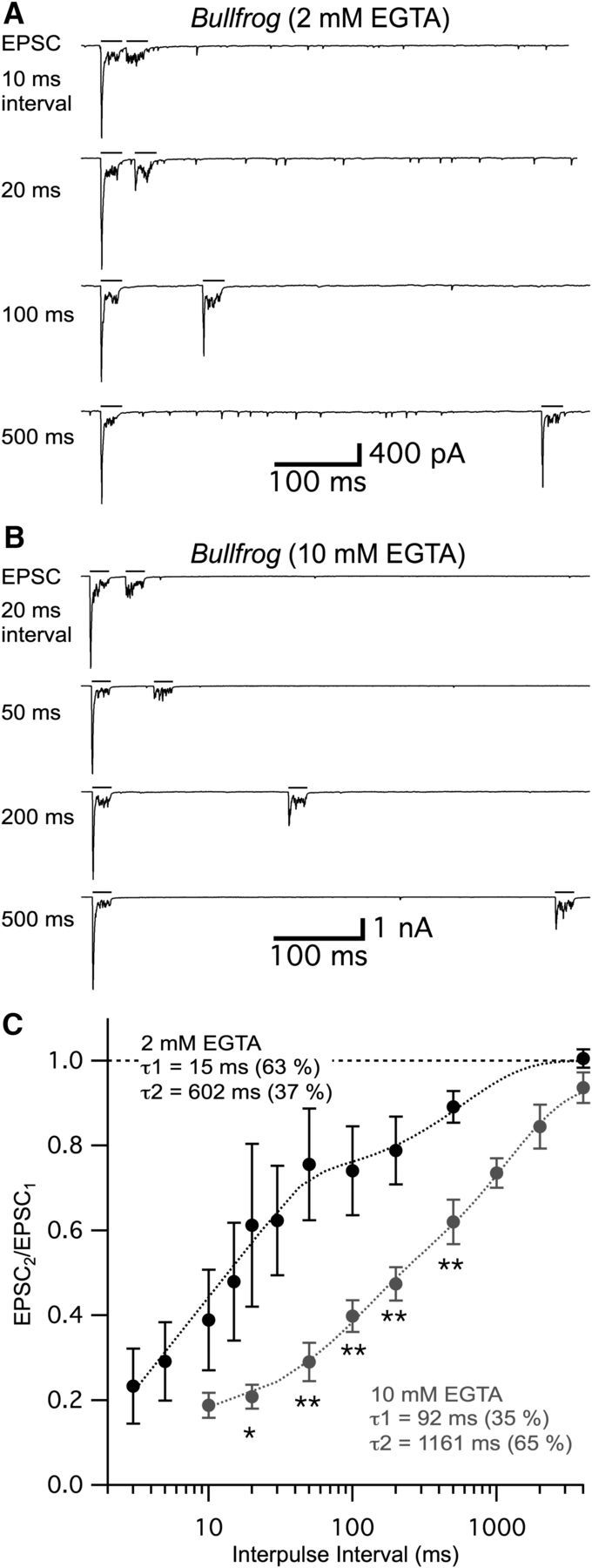
Recovery from paired-pulse depression at bullfrog hair cell synapses is significantly slower with 10 mM EGTA. ***A***, ***B***, EPSCs evoked by two depolarizing pulses were obtained by paired recordings from an afferent fiber and an amphibian papilla bullfrog hair cell. The hair cell was depolarized from −60 mV to −30 mV for 20 ms (black bars) with various IPIs (interpulse intervals). The intracellular Ca^2+^ buffer of the hair cells was 2 mM EGTA (***A***) or 10 mM EGTA (***B***). The first depolarizing pulse still evokes a large phasic EPSC (EPSC_1_) when 10 mM EGTA is present in the hair cell. However, the recovery of the phasic component of the second EPSC (EPSC_2_) was significantly slower with 10 mM EGTA. ***C***, Summary of the PPR (EPSC_2_/EPSC_1_) recovery time course. Two EPSCs were evoked by depolarizing hair cells using a pair of 20 ms pulse with various IPIs. The 2 mM (black, *n* = 4–9 pairs) and 10 mM EGTA (gray, *n* = 5–8) were used as intracellular calcium buffers within hair cells. All the EPSCs were measured after 4 min from the whole-cell break-in to allow for full dialysis with EGTA. Data with 2 mM EGTA were modified from Cho et al. (2011). PPRs with 20, 50, 100, 200, and 500 ms IPIs were significantly different between 2 mM EGTA and 10 mM EGTA. *p < 0.05 (unpaired *t* test). **p < 0.01 (unpaired *t* test).

To study whether the concentration of EGTA can affect short-term plasticity at low-frequency tuned hair cell synapses (tuned to ∼400–500 Hz), we performed paired recordings between adult bullfrog hair cells and their afferent fibers. We held presynaptic hair cells at −60 mV, which is close to their physiological *in vivo* resting membrane potential (Crawford and Fettiplace, 1980; Pitchford and Ashmore, 1987) and measured paired-pulse ratios (PPRs) of EPSCs using 2 and 10 mM intracellular EGTA in the patch pipette of the hair cell (Fig. 7*A*). Hair cells were stimulated by a pair of 20 ms depolarizing pulses from −60 mV to −30 mV with various IPIs and EPSCs recorded from the connected postsynaptic afferent fibers (Fig. 7*A*,*B*). The average amplitude of the first EPSC was not significantly different with 2 mM (2504 ± 307 pA, *n* = 20) and 10 mM EGTA (2582 ± 499 pA, *n* = 18, *p* = 0.89, unpaired *t* test), showing that the RRP exocytosis released by 20 ms pulses is insensitive to the concentration of EGTA. These results using AMPA receptor-mediated EPSCs confirm our earlier results with Δ*C*_m_ changes in hair cells held at −90 mV (see Fig. 5*A*,*C*).

We next studied the changes in PPR with 2 and 10 mM EGTA. While hair cells were held at −60 mV, the second EPSC was smaller than the first EPSC throughout various IPIs (from 3 ms to 4 s), showing a robust paired-pulse depression (Fig. 7*A*,*B*). For short IPIs, such as 20 ms, this synapse shows very strong paired-pulse depression; and as the IPIs get longer, paired-pulse depression recovers (Fig. 7*A*,*B*). This depression is not caused by AMPA receptor desensitization (Graydon et al., 2014), and more likely reflects vesicle pool depletion (Cho et al., 2011). We examined whether the enhanced level of EGTA can affect the recovery from paired-pulse depression. With 2 mM intracellular EGTA, a double exponential function could fit the recovery of PPR with fast (τ_1_ = 15 ms; 63%) and slow (τ_2_ = 602 ms; 37%) time constants (Fig. 7*C*) (Cho et al., 2011). When we increased the concentration of intracellular EGTA to 10 mM, recovery of paired-pulse depression was delayed for both the fast (τ_1_ = 92 ms; 35%) and slow (τ_2_ = 1161 ms; 65%) time constants. The median (weighted-mean) time constant increased significantly from 232 ms with 2 mM EGTA to 787 ms with 10 mM EGTA. These data thus indicate that recovery of paired-pulse depression is dependent on global intracellular Ca^2+^ levels within hair cells. This suggests again that the recruitment of vesicles from a reserve vesicle pool to the RRP is sensitive to the intracellular levels of EGTA and thus to the intracellular free Ca^2+^.

### Exocytosis under physiological resting membrane potential in gerbil IHCs

**Figure 8.**
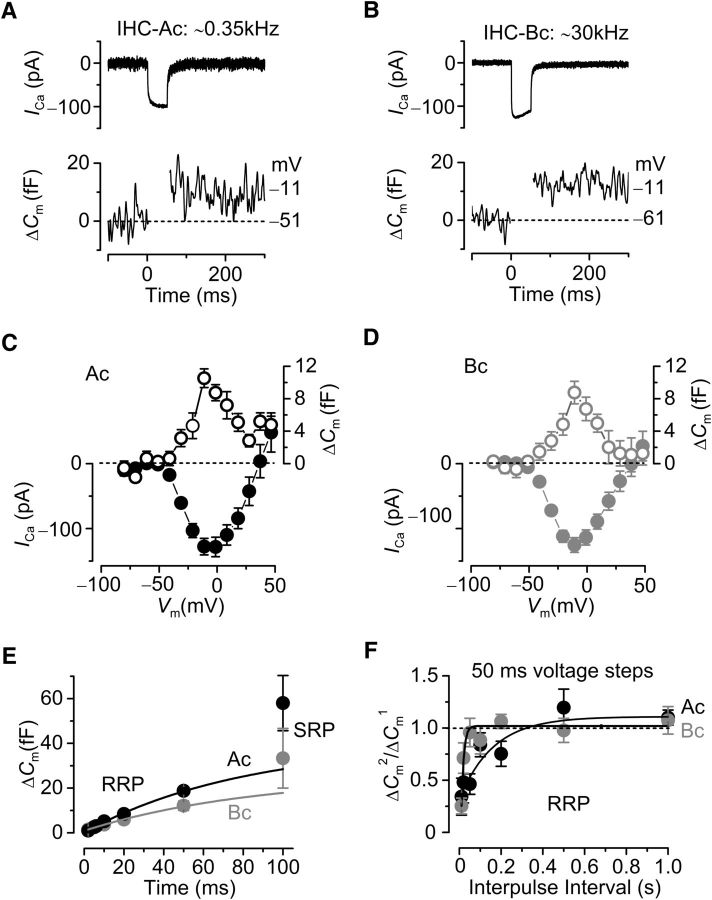
Exocytosis in gerbil IHCs under *in vivo* resting membrane potential. ***A***, ***B***, *I*_Ca_ and ΔC_m_ from apical (***A***) and basal (***B***) IHCs in the presence of 1 mM EGTA in the intracellular solution. Voltage protocol is as described in Figure 1, apart from the holding membrane potential, which was −51 mV for apical and −61 mV for basal IHCs. For clarity, only responses at the resting membrane potential and the peak of *I*_Ca_ (−11 mV) are shown. ***C***, ***D***, Average peak *I–V* and Δ*C*_m_–*V* curves in apical (***C***: P19-P28, *n* = 7) and basal (***D***: P19-P28, *n* = 9) IHCs. ***E***, Average Δ*C*_m_ from apical (black: P19-P28, *n* = 7) and basal (gray: P19-P28, *n* = 5) IHCs obtained in response to voltage steps from 2 ms to 100 s (to −11 mV). Voltage steps up to 50 ms could be fitted by a single exponential function, which reveals the RRP, and values were as follows: apical, maximum Δ*C*_m_ = 40.1 ± 14.9 fF, τ = 81 ± 38 ms; basal, Δ*C*_m_ = 25.6 ± 10.5 fF, τ = 86 ± 42 ms. Voltage steps to 100 ms were able to additionally recruit the SRP. ***F***, Average Δ*C*_m_ ratio (Δ*C*_m_^2^/Δ*C*_m_^1^: see Fig. 6*A*) from apical (black) and basal (gray) IHCs elicited in response to 50 ms depolarizing voltage steps to −11 mV (holding potential of −51 mV for apical and −61 mV for basal IHCs) at time 0 and varying the IPI between 10 ms and 1 s after the initial step. Data were well fitted with a single exponential function. Note that the time on the x-axis in ***E*** indicates the voltage step duration.

The above experiments in mice and gerbils were performed using a holding potential of −81 mV, which is commonly used for exocytosis (capacitance measurements) studies from hair cell ribbon synapses (mouse: Moser and Beutner, 2000; Johnson et al., 2010; Wong et al., 2014; gerbil: Johnson et al., 2009; bullfrog: Li et al., 2009; Cho et al., 2011). Because the estimated *in vivo* resting potential is likely to be ∼−50 mV for apical and −60 mV for basal IHCs (Johnson et al., 2011; Johnson, 2015), and *I*_Ca_ has been shown to activate at ∼−60 mV (gerbils) (Johnson and Marcotti, 2008), cells will be subjected to some continuous Ca^2+^ influx even at rest (see Materials and Methods). Therefore, we investigated gerbil IHC (P19-P28) exocytosis and the coupling between Ca^2+^ influx and the RRP using the more physiological resting membrane potentials (Fig. 8). For these experiments, 1 mM EGTA was used as the intracellular Ca^2+^ buffer in apical and basal IHCs because it produces comparable Δ*C*_m_ (Johnson et al., 2008) as those measured in perforated patch recordings (Fig. 8*E*) for both the RRP and SRP. This also allowed us to test the specific effect of the theoretical *in vivo* membrane potential on the release and replenishment of the RRP. Despite the different resting membrane potentials, the maximal *I*_Ca_ (apical: −128 ± 13 pA, *n* = 7; basal: −125 ± 11 pA, *n* = 9) and the corresponding Δ*C*_m_ (apical: 10.5 ± 1.1 fF; basal: 8.8 ± 1.4 fF) was similar between apical and basal IHCs (Fig. 8*A–D*), as well as the size of the RRP (Fig. 8*E*). However, the rate of Δ*C*_m_ recovery during paired pulses was significantly faster in basal (τ = 27 ± 11 ms, *n* = 6, from fits to single IHCs, *p* < 0.02) than in apical IHCs (τ = 156 ± 43 ms, *n* = 5). This is in line with our findings in the bullfrog showing that faster recovery depends on the availability of global free intracellular Ca^2+^ present in a microdomain situation (Fig. 7*C*).

## Discussion

Using physiological recording conditions, in terms of extracellular Ca^2+^ level and body temperature, we show that the coupling between Ca^2+^ channels and the Ca^2+^ sensor for vesicle fusion changes as a function of the cell's frequency position. While low-frequency hair cells (∼<2 kHz), which phase-lock to sound, exhibit a nanodomain coupling between Ca^2+^ channels and Ca^2+^ sensor, high-frequency cells have a looser coupling, which becomes progressively more microdomain along the gerbil cochlea. We have also shown that the level of intracellular Ca^2+^ buffer affects the speed of recovery from paired-pulse synaptic depression. We propose that either nanodomain or microdomain coupling is present in mature auditory hair cells, the function of which is to preserve the precise temporal coding of sound in phase-locked low-frequency hair cells and stimulus intensity in high-frequency cells, respectively.

### Mechanisms of Ca^2+^ influx-secretion coupling in IHCs

A characteristic feature of the coupling between Ca^2+^ entry and vesicle fusion at IHC ribbon synapses is the change in the Ca^2+^ dependence of exocytosis from a high-order relation in immature cells to linear in mature post-hearing cells (Brandt et al., 2005; Johnson et al., 2005, 2008, 2010; Wong et al., 2014). However, such linearization in synaptic function only occurs in mature high-frequency IHCs responding to sound frequencies above a few kHz (Johnson et al., 2008, 2009), which encompasses the entire frequency range in the mouse cochlea (∼3–100 kHz) (Greenwood, 1990) but only the middle and basal regions in the gerbil (∼0.1–60 kHz) (Müller, 1996). This exocytotic linearization implies that vesicle fusion scales linearly with Ca^2+^ entry, which in mature high-frequency IHCs has been proposed to depend upon the developmental tightening of the spatial coupling between Ca^2+^ channels and vesicle release Ca^2+^ sensors (Ca^2+^-nanodomain coupling) (Brandt et al., 2005; Wong et al., 2014). In this scenario, one or very few Ca^2+^ channels are sufficient to govern the release of a nearby vesicle (Brandt et al., 2005; Graydon et al., 2011; Zampini et al., 2013). However, an alternative hypothesis is that the linearization is due to developmental changes in the Ca^2+^ sensor(s) that affect the intrinsic Ca^2+^ dependence of the synaptic machinery. Although otoferlin is the major Ca^2+^ sensor in IHCs (Roux et al., 2006; Safieddine et al., 2012), synaptotagmin IV is essential for establishing the linear exocytotic Ca^2+^ dependence (Johnson et al., 2010), which could arise from its inability to bind Ca^2+^ in the C2A domain (Südhof, 2002). In this second hypothesis, a Ca^2+^-microdomain coupling scenario could be postulated (Wang and Augustine, 2015). These two synaptic scenarios (i.e., nano- and micro-domain coupling) may indeed coexist within the same auditory organ because low- and high-frequency IHCs are specialized to emphasize mainly the phasic or sustained components of the cell's *in vivo* receptor potential, respectively (Johnson, 2015).

### Nano- versus micro-domain coupling at hair cell ribbon synapses

In squid giant synapses and mature calyx of Held, synaptic vesicle release is reduced more effectively by BAPTA than by EGTA (Augustine et al., 1991; Fedchyshyn and Wang, 2005; Chen et al., 2015; Nakamura et al., 2015), indicating a nanodomain coupling between Ca^2+^ channels and exocytotic Ca^2+^ sensors at mature synapses. However, recent findings have shown that mature hippocampal synapses can also operate via a loose coupling (Vyleta and Jonas, 2014), challenging the view that Ca^2+^-microdomain mode of Ca^2+^ signaling is only a characteristic of immature synapses (e.g., Meinrenken et al., 2002; Fedchyshyn and Wang, 2005; Leão and von Gersdorff, 2009; Wang and Augustine, 2015). Instead, it suggests that the vesicle release modality is optimized for specific functional requirements independent from the stage of cell maturation.

Here we found that the RRP of low-frequency hair cell ribbon synapses, in both the mammalian cochlea (∼350 Hz) and bull frog papilla (<1 kHz), were relatively insensitive to EGTA, whereas RRP release from IHCs responding above a few kHz was either largely reduced (∼2–3 kHz) or abolished (∼30 kHz). This indicates that the spatial coupling between Ca^2+^ influx and exocytosis progressively changes along the gerbil auditory organ to cover a wider hearing range (∼0.1–60 kHz). Although it has previously been reported that the RRP in mouse IHCs was insensitive to 5 mM EGTA (Moser and Beutner, 2000), perhaps due to the use of high unphysiological extracellular Ca^2+^, paired recordings from IHCs and auditory afferent fibers in the rat cochlea have demonstrated that the rate of release (EPSCs/ms) was largely reduced by 5 mM EGTA (Goutman and Glowatzki, 2007).

### Vesicle recruitment and the Ca^2+^ dependence of recovery from depression

We found that apical IHCs tuned to ∼350 Hz in the gerbil recovered fully from paired-pulse depression within ∼200 ms for 50 ms depolarizing pulses and with 0.1 mM EGTA. This recovery rate was slowed by 10 mM EGTA. Similar results were obtained in bullfrog hair cells tuned to lower CF (400–500 Hz; Fig. 7*C*). The enhanced fast recovery of EPSCs with lower Ca^2+^ buffering may be due to the greater spread of Ca^2+^ that speeds the replenishment of vesicles to the ribbon (Van Hook et al., 2014). Recovery from paired-pulse depression was even faster in basal gerbil IHCs (<100 ms), which is consistent with their microdomain coupling. These recovery rates are extremely rapid compared with that in retinal bipolar cells, which also operate via ribbon synapses (Palmer et al., 2003). This rapid recovery may be an evolutionary adaptation for hair cell ribbon synapses, which are specialized to detect rapid sound signals with short gaps and encode these as firing patterns in the auditory nerve.

### Ca^2+^ influx-secretion coupling and hair cell receptor potential

The receptor potential of low-frequency IHCs (up to a few kHz) has a predominantly phasic component that is phase-locked to the sound frequency and graded in size to the stimulation intensity (Dallos, 1985; Cheatham and Dallos, 1993). The localization of low-frequency sound is accomplished by cells in the auditory brainstem that detect minute time delays in the arrival of the phase-locked afferent activity originating from the two ears (∼10 μs) (Grothe et al., 2010). Such a precise temporal coding has to be preserved at IHC ribbon synapses, and the nanodomain coupling scenario would guarantee rapid and reliable vesicle fusion (Neher, 1998). In the low-frequency cochlear region, a nanodomain coupling would also be required to explain why the time delay in vesicle fusion is similar at all levels of IHC depolarization (i.e., stimulus intensity). This property is crucial for the accurate preservation of stimulus timing at all sound intensities and has been suggested to be governed by the single Ca^2+^ channel properties of first latency and current amplitude (Magistretti et al., 2015). At very low frequencies, the relatively slow depolarizing cycle will allow sufficient Ca^2+^ influx to saturate the exocytotic Ca^2+^ sensor, even at low sound intensities. At higher frequencies (up to ∼2 kHz), but still within the limit for phase-locking, the reduced time for Ca^2+^ influx into IHCs is likely to be insufficient to trigger exocytosis; this could be overcome, in a nanodomain scenario, by the elementary Ca^2+^ tail currents, where the amplitude and speed of the current are maximized and constant. Indeed, when sinusoidal stimuli of a few hundred hertz were applied to rat IHCs or hair cells from the bullfrog papilla, afferent fibers responded with large EPSCs that occurred more frequently during the repolarizing phase of the cycles (Goutman, 2012; Li et al., 2014), which corresponded to the Ca^2+^ tail currents.

The filtering characteristics of the hair cell membrane prevents phase-locking ≥2–3 kHz (Palmer and Russell, 1986), so receptor potentials are mainly graded and sustained to represent sound intensity and stimulus envelope (Russell and Sellick, 1978). High-frequency sound localization is performed by cells that compare interaural-level differences originating from graded responses in >3 kHz IHCs of each ear (Caird and Klinke, 1983). Therefore, high-frequency IHCs are not designed to follow the frequency components of sound and, as such, do not require the precise timing provided by nanodomain coupling (Matveev et al., 2011), which is likely to be unsuitable for accurate intensity coding. Instead, the changes in the amplitude and kinetic properties of the macroscopic *I*_Ca_ with sound intensity are now more relevant (Magistretti et al., 2015), which is more in line with a microdomain coupling reported in this study.

### Damage due to loud sounds: why are basal IHCs more susceptible?

We found that high-frequency IHCs (especially those at ∼30 kHz) exhibit a strong block of exocytosis by 10 mM EGTA, indicating that these cells cannot have a large endogenous Ca^2+^ buffering capacity, because it would severely impair transmitter release. This was confirmed by the estimated intracellular Ca^2+^ buffer expressed as an equivalent of EGTA concentration (Fig. 3). Indeed, a triple knock-out mouse for different Ca^2+^-binding proteins did not reveal changes in synaptic sound encoding (Pangršič et al., 2015), suggesting that high-frequency IHCs may thus have a relatively low concentration of Ca^2+^-binding proteins. By contrast, low-frequency tuned bullfrog hair cells have an estimated 8 mM of high-affinity Ca^2+^-binding sites on small mobile proteins (e.g., parvalbumin and calbindin) (Heller et al., 2002), suggesting that their endogenous Ca^2+^ buffering capacity may be more equivalent to 10 mM EGTA.

High-frequency hair cell synapses are also particularly vulnerable to damage during loud noises and aging, which has been shown to lead to the loss of both IHC synaptic ribbons (Kujawa and Liberman, 2009; Kujawa and Liberman, 2015) and low-spontaneous rate afferent fibers (Furman et al., 2013). We thus propose that low-frequency IHCs may express higher concentrations of Ca^2+^-binding proteins, which will not block exocytosis but may confer neuroprotection against excessive Ca^2+^ influx during prolonged stimulation. By contrast, the low Ca^2+^ buffer capacity in high-frequency basal IHCs, which is required for their graded release, will make them more prone to Ca^2+^-induced cytotoxicity. A tonotopic gradient in Ca^2+^-binding protein expression has been reported in auditory hair cells (Hackney et al., 2003, 2005; Patel et al., 2012), which may facilitate a frequency-dependent tuning of exocytosis in some animal species (Schnee et al., 2005; Rutherford and Roberts, 2006; Patel et al., 2012).
